# Lung Ultrasound for the Detection of Radiologic Progression of Interstitial Lung Abnormalities in Patients With Rheumatoid Arthritis

**DOI:** 10.1002/art.70161

**Published:** 2026-05-03

**Authors:** Marie Vermant, Valerie Van Ballaer, Joseph Jacob, Tine Follet, Ryoko Egashira, Kenny Anseele, Heleen Cypers, Laurens J. De Sadeleer, Nico De Crem, Tinne Goos, Barbara Neerinckx, Veerle Taelman, Nathalie Veyt, Alexandros Kalkanis, Patrick Verschueren, Wim A. Wuyts

**Affiliations:** ^1^ Department of Chronic Diseases and Metabolism KU Leuven Leuven Belgium; ^2^ Pulmonology Department UZ Leuven Leuven Belgium; ^3^ Radiology Department UZ Leuven Leuven Belgium; ^4^ Satsuma Laboratory University College London Hawkes Institute, University College London London United Kingdom; ^5^ University College London Respiratory, University College London London United Kingdom; ^6^ Rheumatology Department UZ Leuven Leuven Belgium; ^7^ Department of Development and Regeneration KU Leuven Leuven Belgium

## Abstract

**Objective:**

Interstitial lung disease (ILD) is an important extra‐articular manifestation of rheumatoid arthritis (RA) associated with high mortality and morbidity. Lung ultrasonography (LUS) has recently emerged as a possible screening tool. We assessed the role of LUS in monitoring radiologic progression and the development of new interstitial lung abnormalities (ILAs).

**Methods:**

In this prospective pilot study, patients with RA underwent high‐resolution computed tomography (HRCT), pulmonary function tests (PFTs), symptom assessment, and LUS at baseline and 12 to 18 months later. B‐lines were quantified using a 72‐zone protocol. Radiologic changes across longitudinal HRCTs were scored on a five‐point Likert scale by blinded thoracic radiologists. Six multivariable logistic regression models were built to predict HRCT progression using combinations of LUS parameters, PFTs, symptoms, and clinical characteristics.

**Results:**

Eighty‐one patients completed the follow‐up HRCT, and per‐protocol LUS was performed in 95% (77/81). Radiologic progression was observed in 31% (25/81). An increase in B‐lines was significantly associated with radiologic progression (*P* = 0.018), and a linear trend was confirmed (*P* = 0.026). In multivariable analysis, both baseline B‐lines (*P* = 0.029) and the change in B‐lines over time (*P* = 0.012) significantly predicted progression (area under the curve [AUC] 0.78, 95% confidence interval [CI] 0.64–0.92). When combining LUS and change in diffusing capacity for carbon monoxide (AUC 0.82, 95% CI 0.70–0.94), the model fit remained similar and outperformed symptom scores (AUC 0.58, 95% CI 0.40–0.75, *P* = 0.020) and PFTs (AUC 0.59, 95% CI 0.44–0.74, *P* = 0.022).

**Conclusion:**

LUS may be a useful, radiation‐free tool to monitor and predict radiologic progression of ILAs in RA. These findings support further research into integrating LUS into longitudinal RA‐ILD assessment, particularly as part of a multimodal approach.

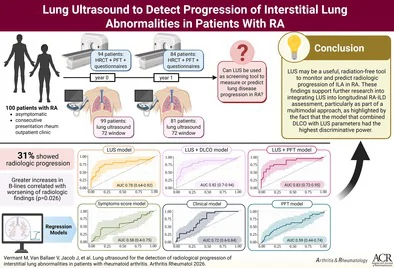

## INTRODUCTION

Interstitial lung disease (ILD) is a severe and potentially life‐threatening extra‐articular manifestation of rheumatoid arthritis (RA) linked to significant morbidity and increased mortality.^1^, ^2^, ^3^, ^4^, ^5^ Although prevalence estimates of RA‐ILD vary widely, approximately 10% of patients with RA develop clinically relevant ILD at any point during the disease course.^6^, ^7^, ^8^ The presence of ILD could guide the selection of a disease‐modifying antirheumatic drug (DMARD). For example, methotrexate has been shown to have a protective effect on the development of fibrotic ILD.^9^ Similarly, there are promising but early results for rituximab and abatacept in the control of ILD.^10^

RA‐associated ILD encompasses a heterogeneous spectrum of disease, ranging from interstitial lung abnormalities (ILAs)^11^, ^12^ to clinically overt, progressive ILD. Only a subset develops a progressive phenotype. Several frameworks have been proposed to define progression in fibrotic ILD, including the INBUILD and American Thoracic Society (ATS) criteria, which integrate changes in lung function, symptoms, and radiologic extent of fibrosis over time.^13^, ^14^ Detection of progression is vital to tailor DMARD therapy and to assess the necessity to timely initiate antifibrotic therapy.

Repeated high‐resolution computed tomography (HRCT) scans, therefore, currently play a pivotal role in detecting new ILAs and in determining radiologic progression. However, because HRCT scans are associated with radiation, frequent use could potentially increase the risk of malignancy.^15^ Furthermore, HRCT is associated with a relatively high cost, and availability can be limited. This raises the question of whether an alternative diagnostic tool could be used as a proxy for radiologic progression and monitoring to increase the pretest probability for HRCT.

Lung ultrasonography (LUS) is a radiation‐free tool and is associated with lower costs than CT.^16^ It can be performed both at the bedside and in the outpatient clinic. Recently, multiple studies have assessed the role of LUS in the early detection of RA‐ILD in patients with RA.^17^, ^18^, ^19^, ^20^, ^21^ LUS can be used to assess both pleural and parenchymal abnormalities.^22^ In RA‐ILD, as well as in other interstitial syndromes, such as pulmonary edema or viral infections, B‐lines are the main ultrasonographic finding. B‐lines are vertical, comet tail–like artifacts that expand across the whole length of the screen and move together with the lung parenchyma during breathing.^23^ Although no consensus exists yet on the optimal scanning protocol to determine the exact number of B‐lines suggestive of underlying pathology like ILD, LUS appears to be a sensitive screening tool, with a high negative predictive value.^17^, ^18^, ^19^, ^20^, ^21^ However, its role in monitoring radiologic progression of ILD and in detecting new ILAs remains unexplored, and no studies to date have systematically evaluated longitudinal changes in LUS findings in relation to radiologic progression in patients with RA.

The current study aimed (1) to assess the correlation between the change in the number of B‐lines over time and the presence of radiologic progression on HRCT; (2) to evaluate whether LUS parameters, namely the change in the number of B‐lines over time and the number of B‐lines at baseline, could predict progression of ILAs; and (3) to compare these LUS parameters with changes in clinical symptoms (symptom‐based scores) and pulmonary function tests (PFTs; forced vital capacity [FVC] and diffusing capacity for carbon monoxide [DLco]) as predictors of progression.

## METHODS

### Study design

This prospective study included 100 adult patients who presented at the University Hospitals Leuven outpatient rheumatology clinic between January 2023 and November 2023. All patients had a previously confirmed diagnosis of RA by a team of expert rheumatologists (PV, BN, VT, and HC), guided by the American College of Rheumatology/EULAR classification criteria.^1^ Patients consecutively presenting to the outpatient clinic were randomly selected for this study. Exclusion criteria consisted of an uncertain diagnosis of RA and incapability of giving informed consent. A flow diagram of patient inclusion and the availability of imaging data is shown in Supplementary Figure 1.

All included patients underwent HRCT, PFT, and LUS at baseline (baseline time point) and at a follow‐up (one‐year time point), which was scheduled 12 to 18 months after baseline technical examinations. The initial PFT included spirometry and diffusion testing and was performed within three months after inclusion. At the one‐year time point, all tests were scheduled and performed on the same day. Additionally, all patients answered several questionnaires concerning their pulmonary and rheumatologic symptoms at baseline and at the one‐year time point. In the routine care for patients with RA, the Health Assessment Questionnaire and visual analog scales (VAS) for patient global assessment, pain, and fatigue are collected. To evaluate the respiratory symptoms, additional per‐protocol study questionnaires were collected to assess the presence of dyspnea and cough: the modified Medical Research Council (mMRC) dyspnea scale and the VAS for cough (VAS cough).

Ethical approval was obtained through the Ethical Committee of the University Hospitals Leuven (S66851) in November 2022. The baseline characteristics of this patient group have been described in previous publications regarding this study project (“;Implementation of a multimodal screening program for interstitial lung disease in rheumatoid arthritis patients”, SCAR).^20^, ^22^ Written informed consent was obtained from all participants. Patient experts were not involved in trial design.

### LUS

LUS was performed with a curved 3.5‐MHz array probe using a Philips Lumify portable ultrasound system. Two experienced examiners (MV and TF), trained through the European Respiratory Society, executed all ultrasound examinations. Lung‐specific presets were used, with harmonic imaging disabled and postprocessing (“reject”) minimized. All examinations were executed in a seated position, using the 72‐zone lung intercostal space (IS) protocol.^24^ Per IS, the total number of B‐lines was counted. If they were confluent, a previously described semiquantitative method was used.^25^, ^26^ The position and the total scoring of the artifacts were recorded using our in‐house developed Android application.^22^ Scorers were blinded to HRCT and PFT findings during the examination.

To assess the interobserver and intraobserver variability, 110 random three‐second ultrasound clips were collected and scored for the number of B‐lines (0–10) by the two independent scorers (MV and TF). Cohen's kappa interrater agreement was calculated for the presence and number of B‐lines for both interobserver and intraobserver variability. Additionally, an intraclass correlation coefficient (ICC) was calculated for the number of B‐lines.^27^ Furthermore, we calculated the Pearson correlation coefficient between the change in B‐lines and the number at baseline to assess for an underlying correlation.

### HRCT scan scoring

Noncontrast HRCT, including some photon‐counting CT scans (depending on clinical availability) were acquired at baseline and follow‐up (time point 1). All scans were performed using an HRCT protocol. Image acquisition was obtained with a maximum slice thickness of 1 mm (minimum 0.4 mm for photon‐counting CT and 1 mm for energy‐integrating detector CT), with patients positioned supine and imaged at full inspiration. All examinations were compared with prior chest CT studies acquired using identical acquisition parameters to assess temporal evolution of imaging findings. Additional acquisitions, including prone and expiratory imaging, were not scored and/or compared in the present study. Two ILD radiologists (JJ and VVB) independently reviewed the CT scans for CT quality and for the presence of ILAs. A previously described qualitative score (no ILA, nonfibrotic ILAs, fibrotic ILAs, and undiagnosed ILD)^28^ was assigned to all scans. All scans were pseudonymized and temporally randomized before scoring to prevent bias of scan sequence. ILA progression was rated on a five‐point Likert scale by the same two radiologists (JJ and VVB) as follows: much worse, −2; worse, −1; no change, 0; better, 1; and much better, 2. Radiologic progression was defined as worsening or new development of ILAs on HRCT. Interobserver variability was calculated using Cohen's kappa interrater agreement correlation, and squared weights were applied to account for the magnitude of disagreement between raters. This method penalizes more severe discordance more heavily, thus aligning with the ordinal nature of the data. The radiologists were blinded to clinical data and each other's scores.

For the ILA progression parameter, a consensus score was obtained. If both radiologists agreed, the value was withheld. In cases in which both scored in the same direction (eg, −2 and −1 or 1 and 2), an average value (–1.5 or 1.5) was assigned. When the direction of change (improvement, worsening, or stability) was disputed, a third radiologist (RE) scored the scan using the same Likert scale, and the consensus score of the three readers was retained. Before analysis of the ILA progression parameter, all ordinal scores were explicitly recoded as ordered factors after reassigning scans to their correct chronological time points (baseline and one‐year time point) following temporal randomization.

### Statistical analysis

All analyses were performed in R (version 4.5.0). An α level of 0.05 was used. There was no formal sample size calculation because this was a pilot study. Missingness was assessed using Little's missing completely at random (MCAR) test to assess whether the data were MCAR. If the MCAR assumption was not rejected, and due to the exploratory setup of this trial, missing data were removed from the analyses. Furthermore, given the exploratory nature of this study, *P* values were not adjusted for multiple comparisons and should be interpreted cautiously.

#### Demographics

The trial was conceptualized as a pilot study. Normality of the data was assessed using the Shapiro–Wilk test. Nonnormal data are reported as median and interquartile range. Normal data are reported as the mean and SD. Comparisons between patients who showed radiologic progression and the rest of the study cohort were performed with Fisher's exact test and the Mann–Whitney U test. RA therapy at baseline was categorized (eg, conventional synthetic DMARDs, biologic DMARDs [bDMARDs], bDMARDs + conventional synthetic DMARDs, etc). Due to small numbers per category, individual treatment effects were evaluated descriptively and using Fisher's exact test rather than formal regression modeling.

#### Exclusion of two data points

Two patients were excluded from the analysis. The first underwent major thoracic surgery after the rupture of a thoracic aneurysm during the follow‐up. For the second, the ultrasound quality was deemed insufficient by both ultrasonographers for a correct interpretation.

#### Correlation between ILA progression and B‐line difference

A Kruskal–Wallis rank sum test was used to assess the correlation between the change in B‐lines between the two time points and the ILA progression parameter. We used the ILA progression parameter to group the cases into the following categories: improvement of imaging (score of 1, 1.5, or 2), stable imaging, and worsening of imaging (score of −1, −1.5, or −2). The Kruskal–Wallis rank sum test was also used to assess the correlation between the change in B‐lines between the two time points and these three categories. If a significant result was obtained with a Kruskal–Wallis test, we performed a pairwise comparison using a Wilcoxon rank sum test with a correction for multiple testing via the Benjamini and Hochberg false discovery rate.

Additionally, changes in total B‐line counts between baseline and follow‐up LUS were categorized to facilitate comparison with clinical progression. Patients were assigned to one of seven B‐line change categories based on the absolute difference in total B‐lines: more than 20 fewer (< −20), 10 to 20 fewer (−20 to −11), 6 to 10 fewer (−10 to −6), stable (−5 to 5), 6 to 10 more (6 to 10), 10 to 20 more (11 to 20), and more than 20 more (>20). No validated cutoff for a clinically meaningful B‐line change currently exists; categories were defined a priori to facilitate ordinal analysis. These categories were treated as an ordinal factor for visualization and cross‐tabulation. The association between B‐line change category and progression status (worsened, stable, improved) was assessed using Fisher's exact test. A Cochran–Armitage test was performed to assess the presence of a linear trend over ordered categorical data. A bar plot showing the distribution of B‐line change categories within each progression group was generated. Additionally, per B‐line change category, an odds ratio was calculated for the presence of progression.

#### Multivariable logistic regression

We built six multivariable logistic regression models to predict the presence of progression on CT at one year (worse/not worse) while using the following variables: (1) LUS model: B‐lines at baseline and the change in B‐lines from baseline to one year; (2) symptoms score model: the change in mMRC dyspnea scale and VAS cough scores; (3) PFT model: the change in predicted FVC (FVC%pred) and predicted DLco (DLco%pred); (4) clinical model: a combination of clinical risk factors, namely sex, age, Disease Activity Score in 28 joints using the C‐reactive protein level (DAS28‐CRP), and presence of anti–cyclic citrullinated peptide (anti‐CCP) antibodies; (5) LUS + DLco model: B‐lines at baseline, the change in B‐lines, and the difference in DLco%pred; and (6) LUS + PFT model: B‐lines at baseline, change in B‐lines, and the difference in FVC%pred and DLco%pred. All differences were calculated between baseline and time point 1. Progression for a model was defined as a negative score on the Likert scale (−1, −1.5, or −2). For each model, an area under the curve (AUC) was calculated to assess its predictive power in the assessment of progression. Comparative C‐statistics using a bootstrapping method (n = 5,000) were performed to evaluate differences between the obtained AUCs.^29^ Because the models were exploratory, no corrections for multiple testing were used. Variance inflation factors (VIFs) were calculated to assess the presence of multicollinearity among predictors.

### Data availability

The data underlying this article will be shared on reasonable request to the corresponding author.

## RESULTS

### Demographics

Eighty‐one of the 95 patients who underwent a baseline CT scan underwent a follow‐up CT scan at time point 1 (Table 1). Little's MCAR test indicated that missingness in follow‐up variables (age, sex, PFT parameters, CT findings, respiratory questionnaires) was not systematically related to other observed variables, consistent with MCAR, both in the entire cohort (N = 100; *P* = 0.442) and in the subset of patients with a baseline CT scan (n = 95; *P* = 0.518). Therefore, no adjustments for missingness were made in the analyses. Most patients reported that the combination of the study‐related technical examinations performed, namely the CT scan, PFTs, questionnaires, and LUS, caused a significant burden because they were very time consuming.

**Table 1:** Population characteristics at baseline (N = 81)*

Characteristic	Overall (N = 81)	Not worse (n = 56)	Worse (n = 25)	*P* value^a^
Female	49.0 (60.5)	36.0 (64.3)	13.0 (52.0)	0.3
Seropositive	69.0 (85.2)	48.0 (85.7)	21.0 (84.0)	>0.9
Rheumatoid factor	57.0 (71.3)	39.0 (69.6)	18.0 (75.0)	0.8
ACPA positive	63.0 (80.8)	45.0 (83.3)	18.0 (75.0)	0.5
Ever smoker	44.0 (54.3)	28.0 (50.0)	16.0 (64.0)	0.11
Active smoker	11.0 (13.6)	5.0 (8.9)	6.0 (24.0)	0.085
Treatment at inclusion				0.7
csDMARD	32.0 (39.5)	22.0 (39.3)	10.0 (40.0)	
csDMARD + bDMARD	29.0 (35.8)	22.0 (39.3)	7.0 (28.0)	
bDMARD	8.0 (9.9)	5.0 (8.9)	3.0 (12.0)	
csDMARD + tsDMARD	5.0 (6.2)	2.0 (3.6)	3.0 (12.0)	
GC + csDMARD	5.0 (6.2)	3.0 (5.4)	2.0 (8.0)	
GC	1.0 (1.2)	1.0 (1.8)	0.0 (0.0)	
No treatment	1.0 (1.2)	1.0 (1.8)	0.0 (0.0)	
Ever use of methotrexate	80.0 (98.8)	55.0 (98.2)	25.0 (100.0)	>0.9
Presence of ILD/ILA at baseline				**<0.001**
No ILD	66.0 (81.5)	53.0 (94.6)	13.0 (52.0)	
ILA	5.0 (6.2)	2.0 (3.6)	3.0 (12.0)	
ILD	10.0 (12.3)	1.0 (1.8)	9.0 (36.0)	
Age at inclusion, y	60.5 (53.7–70.7)	57.8 (49.9–66.8)	67.5 (59.5–73.5)	**0.003**
Age at RA diagnosis, y	46.2 (37.3–57.1)	45.2 (34.7–55.8)	51.6 (43.4–64.5)	**0.045**
Disease duration, y	10.4 (4.0–20.3)	9.4 (3.2–20.4)	14.5 (4.5–19.6)	0.5
DAS28‐CRP	2.1 (1.7–3.6)	2.1 (1.7–3.6)	2.1 (1.8–3.0)	0.9
VAS pain	40.0 (10.0–60.0)	35.0 (10.0–60.0)	50.0 (20.0–70.0)	0.3
VAS fatigue	50.0 (15.0–70.0)	50.0 (12.5–70.0)	50.0 (20.0–70.0)	>0.9
mMRC	0.0 (0.0–1.0)	0.0 (0.0–1.0)	0.0 (0.0–1.0)	>0.9
VAS cough	1.0 (0.0–20.0)	0.0 (0.0–10.0)	7.5 (0.0–20.0)	0.4
DLco%pred baseline	90.0 (80.0–97.5)	92.0 (84.0–98.0)	80.0 (63.0–96.0)	**0.004**
FVC%pred baseline	104.0 (96.5–118.0)	105.0 (96.0–120.0)	103.0 (97.0–114.0)	0.6

* Baseline characteristics for the entire population, the population of patients whose CT scans did not progress, and the population of patients whose CT scans did show signs of progression. Values are presented as n (%) for sex, seropositivity, smoking status, treatment, and presence of ILD/ILA and as medians with interquartile ranges (Q1–Q3) for all other variables. Bold values indicate *P* < 0.05. ACPA, anti–citrullinated protein antibody; bDMARD, biologic DMARD; csDMARD, conventional synthetic DMARD; CT, computed tomography; DAS28‐CRP, Disease Activity Score in 28 joints using the C‐reactive protein level; DLco%pred, predicted diffusing capacity for carbon monoxide; DMARD, disease‐modifying antirheumatic drug; FVC%pred, predicted forced vital capacity; GC, glucocorticoids; ILA, interstitial lung abnormalities; ILD, interstitial lung disease; mMRC, modified Medical Research Council dyspnea scale; RA, rheumatoid arthritis; tsDMARD, targeted synthetic DMARD; VAS, visual analog scale.

^a^ Fisher's exact test or Wilcoxon rank sum test.

The majority of the cohort were female seropositive patients in remission (median DAS28‐CRP of 2.1). RA duration was highly variable, and nearly all patients (98.8%) had been treated with methotrexate before inclusion. Of patients, 12.3% (10/81) received a clinical diagnosis of ILD at baseline and were referred for further workup at the pulmonology outpatient clinic. Additionally, 6.2% of patients were diagnosed with ILAs at inclusion for this study. Twenty‐five (30.9%) patients had progression of their interstitial abnormalities or developed new interstitial abnormalities on HRCT. HRCT was stable in 48 (59.2%) patients, whereas in 8 (9.9%) patients, their radiologic ILA features improved. Baseline characteristics of patients who did show signs of radiographic progression compared to patients who did not show progression on HRCT are also shown in Table 1. No significant association was observed between baseline treatment category and ILA progression (Fisher's exact test, *P* = 0.66), but all subgroups were small.

A per‐protocol LUS was performed in 95% of patients (77/81). These missing data were MCAR (*P* = 0.722). Missed examinations (4/81 or 5%) were due to patient‐reported time constraints or operator unavailability.

### Interobserver variability

#### HRCT

Interobserver agreement for scoring the baseline HRCT scan was moderate to substantial (κ = 0.634), and for the second HRCT scan (before rerandomization), it was moderate (κ = 0.538). Agreement regarding the progression of ILAs was substantial (κ = 0.683).

#### LUS

Interobserver agreement between the two scorers (MV and TF) for the 110 short videos was excellent for the presence of B‐lines (κ = 0.94) and substantial for the exact number of B‐lines (κ = 0.689). Intraobserver agreement was excellent for scorer 1 (MV) for both the presence (κ = 0.98) and number of B‐lines (κ = 0.869). For scorer 2 (TF), intraobserver agreement was also excellent for presence (κ = 0.94) and substantial for the number of B‐lines (κ = 0.731). The ICC for interobserver agreement on the number of B‐lines was 0.977 (95% confidence interval [CI] 0.914–0.994). MV performed all baseline LUS. At the one‐year time point, MV performed 74% (57/77) and TF performed 26% (20/77).

### Correlation between radiologic ILA progression and B‐line difference

First, we assessed the relationship between the absolute change in B‐lines between baseline and follow‐up and radiologic ILA progression, as measured by the Likert scale. Although a trend was present, the Kruskal–Wallis test did not reach statistical significance (χ^2^ = 8.41, *P* = 0.135; Figure 1A). When ILA progression was grouped into three categories (worsened, stable, or improved) and we reassessed the correlation, the association was statistically significant (χ^2^ = 8.02, *P* = 0.018; Figure 1B). Patients with progression on HRCT exhibited greater increases in B‐lines over time compared to those with stable radiologic findings (*P* = 0.045). There was a very weak correlation between the number of B‐lines at baseline and changes in the number of B‐lines over time (r = −0.19).

**Figure 1:**
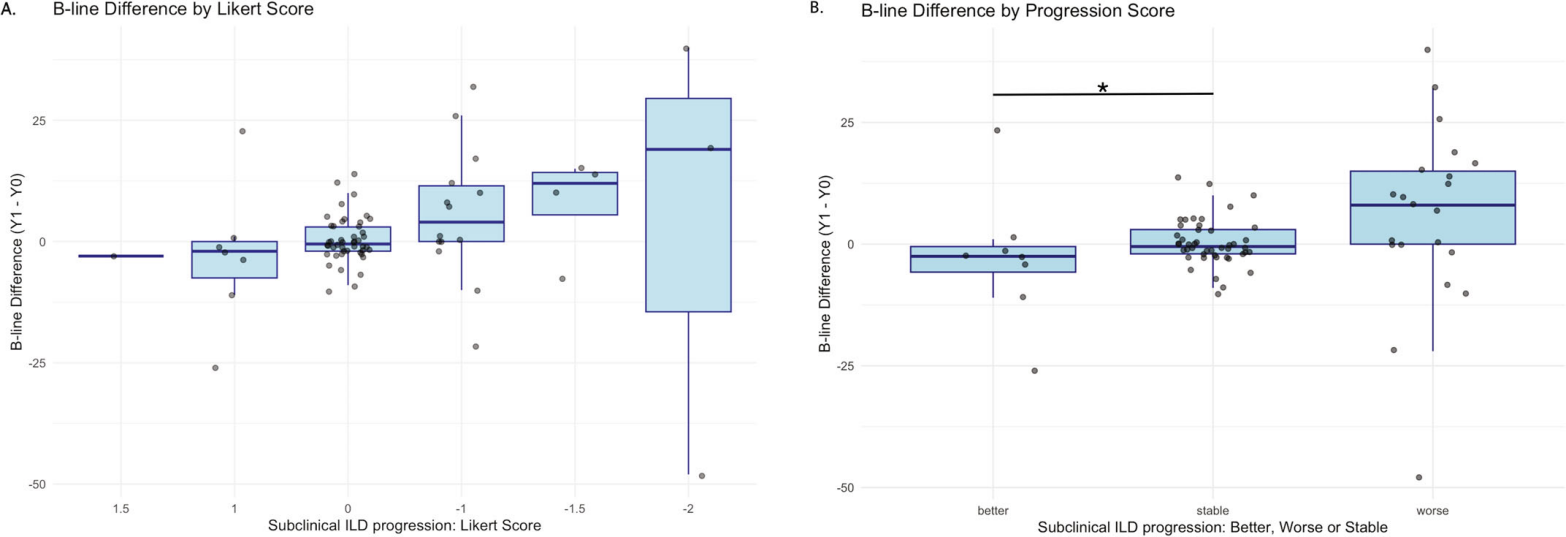
Box plots showing the correlation between interstitial lung abnormality (ILA) progression on computed tomography (CT) and B‐line change. (A) Correlation between ILA progression as expressed per Likert score and the change in the number of B‐lines. Box plots show the difference in the number of B‐lines between time point 1 and baseline per Likert score. A more negative Likert score was defined as more progression, whereas a more positive Likert score was defined as more improvement. (B) Correlation between ILA progression on CT, expressed as worse, stable, or better, and the difference in the number of B‐lines. Box plots show the difference in the number of B‐lines between time point 1 and baseline per Likert progression category (worse, stable, or better). Worse was defined as a negative Likert score for ILA progression (−1, −1.5, or −2). Stable was defined as a Likert score of 0. Better was defined as a positive Likert score for ILA progression (+1, +1.5, or +2). Differences between groups were assessed using pairwise Wilcoxon rank sum tests. Significance is indicated as *P < 0.05; **P < 0.01; ***P < 0.001. Correction for multiple testing was performed (Benjamini and Hochberg false discovery rate). ILD, interstitial lung disease.

A bar chart (Figure 2) illustrates the proportional distribution of B‐line change as a categorical value across the three ILA progression groups (worse, stable, improved). Fisher's exact test confirmed a significant association between B‐line change category and ILA progression (*P* < 0.0001). Furthermore, a Cochran–Armitage trend test supported the presence of a linear trend (*P* = 0.026), with greater increases in B‐lines correlating with worsening of radiologic findings.

**Figure 2:**
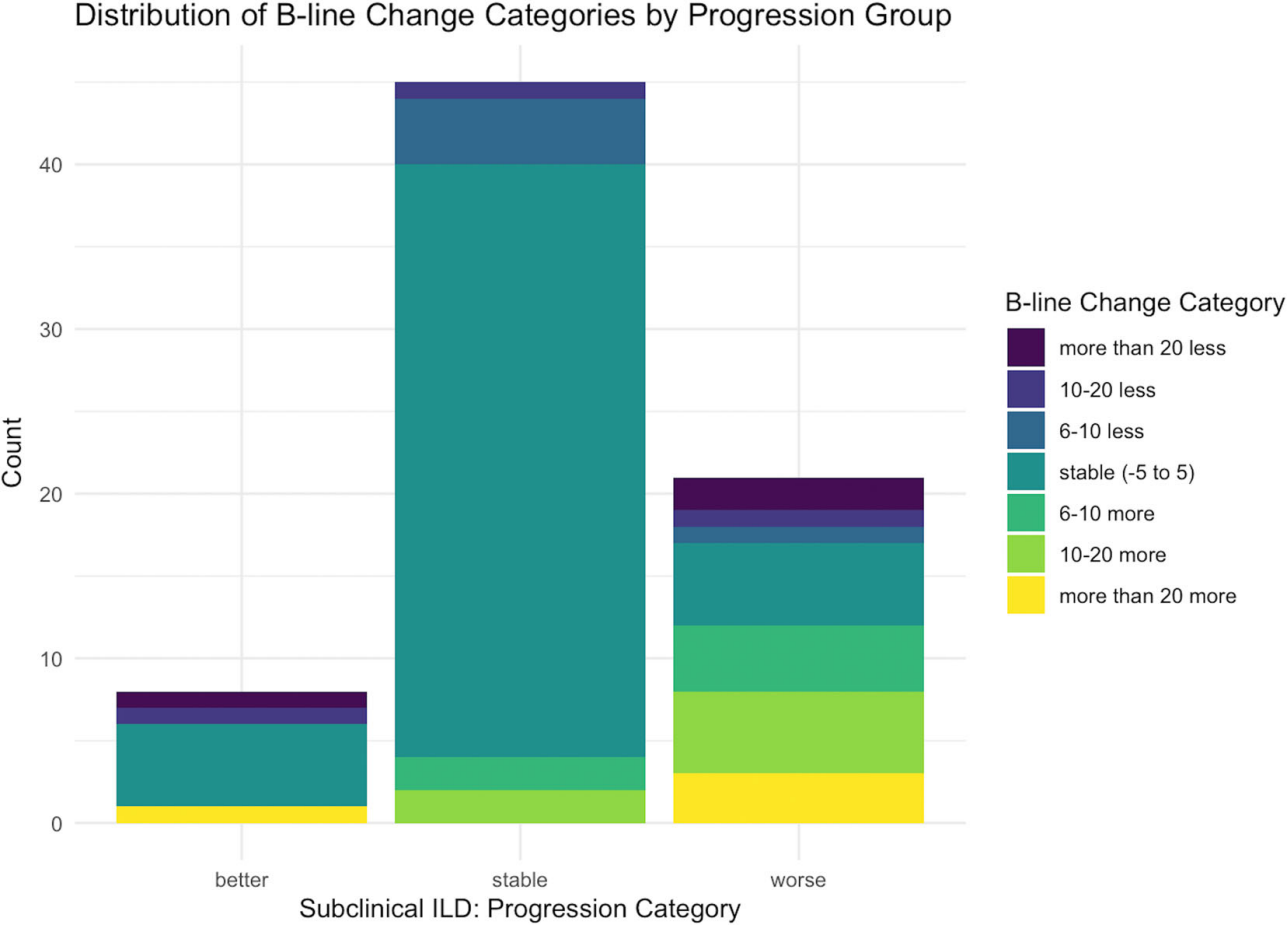
Distribution of change in B‐lines as a categorized variable, expressed as progression group. Distribution of B‐line change categories across interstitial lung abnormality (ILA) progression groups (worse, stable, or improved). Worse was defined as a negative Likert score for ILA progression (−1, −1.5, or −2). Stable was defined as a Likert score of 0. Better was defined as a positive Likert score for ILA progression (+1, +1.5, or +2). Fisher's exact test showed a significant association between B‐line change category and ILA progression (*P* < 0.0001). A Cochran–Armitage trend test indicated a significant linear trend (*P* = 0.026), with greater increases in B‐lines correlating with worsening radiologic outcomes. ILD, interstitial lung disease. Color figure can be viewed in the online issue, which is available at http://onlinelibrary.wiley.com/doi/10.1002/art.70161/abstract.

To quantify this association, odds ratios were calculated for radiologic progression using the stable B‐line category (n = 46) as the reference. Although CIs were wide due to small subgroup sizes, increases in the number of B‐lines were generally associated with higher odds of radiologic worsening. Patients with an increase of 6 to 10 B‐lines had an odds ratio of 8.63 (95% CI 0.97–90.8; n = 6). An increase of 10 to 20 B‐lines yielded an odds ratio of 26.9 (95% CI 3.14–600; n = 7), and an increase of more than 20 B‐lines had an odds ratio of 5.27 (95% CI 0.175–175; n = 4) for CT progression.

In contrast, decreases in B‐line counts were not associated with a significant effect. Patients with a decrease of more than 20 B‐lines had an odds ratio of 2.77 (95% CI 0.07–113; n = 3). Those with a decrease of 10 to 20 B‐lines had an odds ratio of 1.95 (95% CI 0.07–28.5; n = 3), and those with a decrease of 6 to 10 B‐lines had an odds ratio of 1.47 (95% CI 0.06–14.9; n = 5). Figure 3 shows four examples of concordance and discordance between CT‐graphic findings and LUS findings.

**Figure 3:**
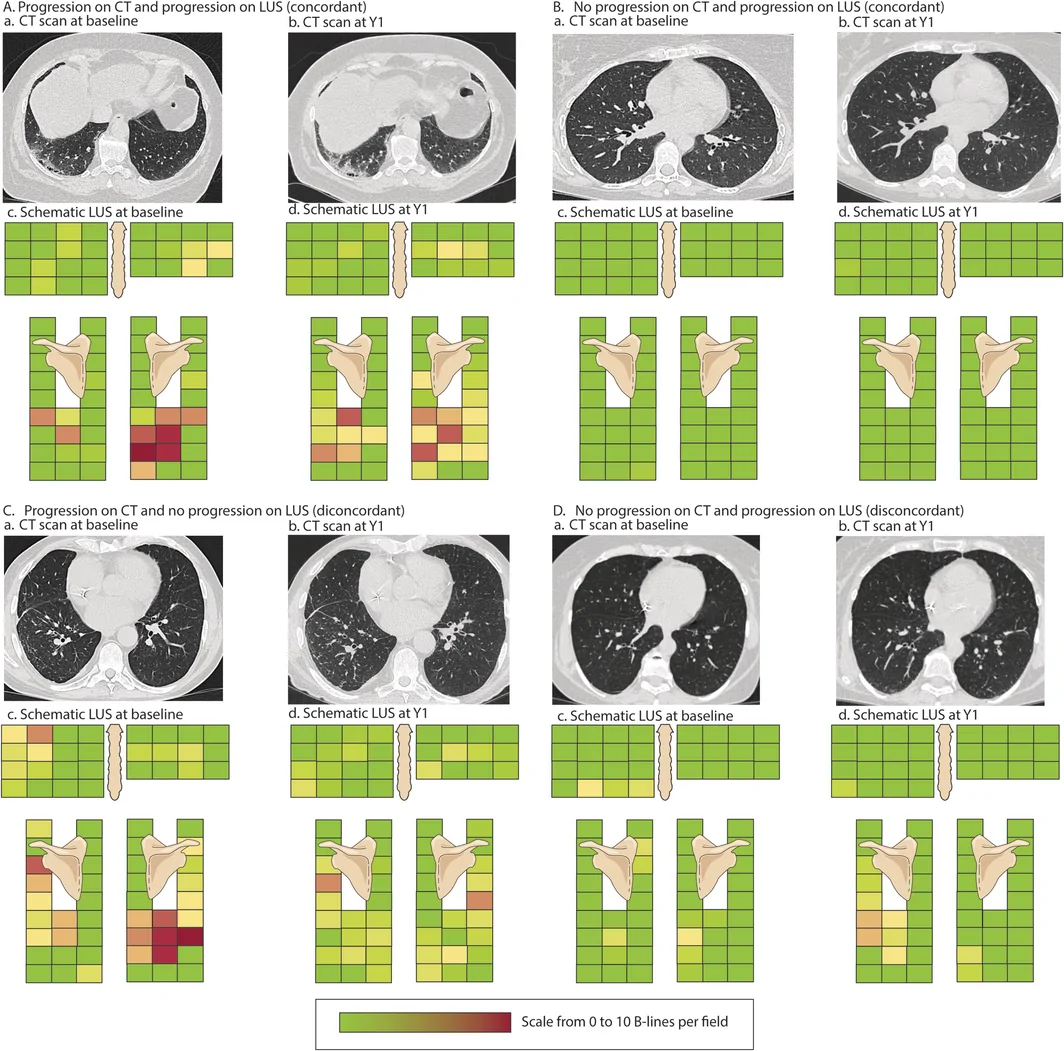
Examples of CT progression and concordance or discordance with LUS progression. Examples of axial HRCT images at baseline (A.a, B.a, C.a, D.a) and at one year (A.b, B.b, C.b, D.b) are shown alongside corresponding schematic LUS heatmaps for patients with and without radiographic progression. Each heatmap represents a standardized 72‐zone LUS scanning protocol, with individual squares corresponding to predefined lung zones. Squares are color coded according to the maximum number of B‐lines detected per zone (range 0–10), with increasing color intensity indicating a higher B‐line count. Panels A and B illustrate cases with LUS–HRCT concordance (absence or presence of progression on both modalities), whereas panels C and D illustrate LUS–HRCT discordance (progression detected on one modality but not the other). In patient A (HRCT score −1.5), the total number of B‐lines increased from 97 at baseline to 111 at one year. In patient B (HRCT score 0), the total number of B‐lines remained stable (1 at both time points). In patient C (HRCT score −2), the total number of B‐lines decreased from 137 at baseline to 89 at one year, whereas in patient D (HRCT score 0), the total number increased from 27 to 35 over one year. Coronary calcifications are visible on HRCT in patients C and D. HRCT, high‐resolution computed tomography scan; LUS, lung ultrasonography. Color figure can be viewed in the online issue, which is available at http://onlinelibrary.wiley.com/doi/10.1002/art.70161/abstract.

### Multivariable logistic regression

Figure 4 shows the receiver operating characteristic curves of the six obtained models and their respective AUCs. In multivariable logistic regression using the LUS model, both baseline B‐lines (*P* = 0.029) and change in B‐lines (*P* = 0.012) were significantly associated with ILA progression, with a model AUC of 0.78 (95% CI 0.64–0.92). The symptoms score model showed no significant associations (mMRC dyspnea scale difference *P* = 0.67, VAS cough difference *P* = 0.24) and had a poor model fit (AUC 0.58 95% CI 0.40–0.75). Similarly, the PFT‐only model (DLco%pred and FVC%pred) showed no significant predictors (DLco%pred *P* = 0.69, FVC%pred *P* = 0.28), with a poor AUC of 0.59 (95% CI 0.44–0.74). The clinical model (sex, age, DAS28, anti–citrullinated protein antibody) identified age as the only significant predictor (*P* = 0.013), with an AUC of 0.72 (95% CI 0.60–0.84). Combining LUS variables with DLco (AUC 0.82, 95% CI 0.70–0.94) or with both DLco and FVC (AUC 0.83, 95% CI 0.72–0.95) resulted in the best‐performing models, with B‐lines at baseline and the difference in B‐lines as significant predictors, whereas pulmonary function parameters remained nonsignificant. Odds ratios for the LUS + DLco and LUS + PFT models are presented in Table 2. All VIF values were less than 2 (range 1.0–1.2), indicating no relevant multicollinearity.

**Figure 4:**
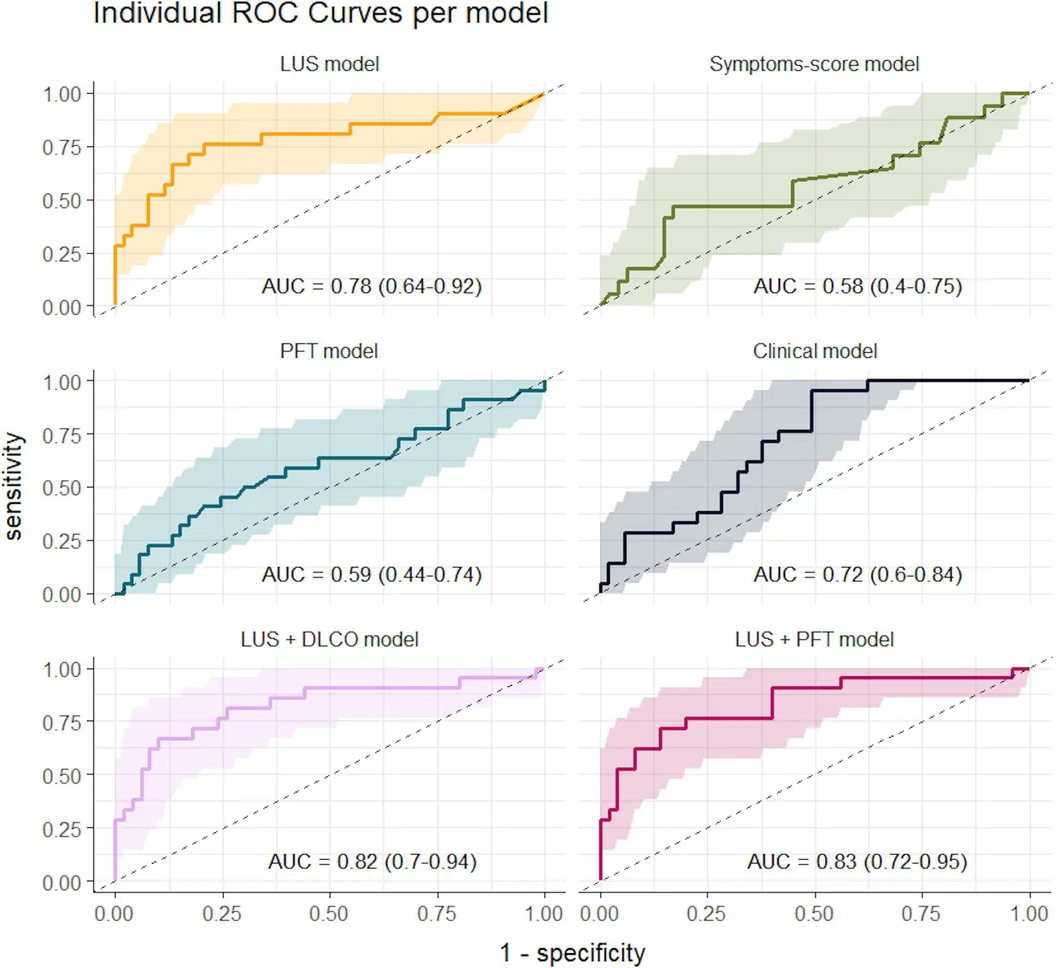
Multivariable logistic regression ROC curves for the detection of radiologic progression based on the following models: (top left) LUS model, based on baseline B‐lines and the difference in B‐lines; (top right) symptoms score model, based on differences in VAS cough and mMRC scores; (middle left) PFT model, based on differences in FVC%pred and DLco%pred; (middle right) clinical model, combination of sex, age, DAS28‐CRP, and presence of anti‐CCP antibodies; (lower left) LUS + DLco model, based on baseline B‐lines, the change in B‐lines, and the change in DLco%pred; and (lower right) LUS + PFT model, based on baseline B‐lines, the change in B‐lines, FVC%pred, and DLco%pred. AUCs for each are given with 95% confidence intervals. Progression was defined as a negative Likert score for interstitial lung abnormality progression (−1, −1.5, or −2). anti‐CCP, anti–cyclic citrullinated peptide; AUC, area under the curve; DAS28‐CRP, Disease Activity Score in 28 joints using the C‐reactive protein level; DLco%pred, predicted diffusing capacity for carbon monoxide; FVC%pred, predicted forced vital capacity; LUS, lung ultrasonography; mMRC, modified Medical Research Council dyspnea scale; PFT, pulmonary function test; ROC, receiver operating characteristic; VAS, visual analog scale. Color figure can be viewed in the online issue, which is available at http://onlinelibrary.wiley.com/doi/10.1002/art.70161/abstract.

**Table 2:** ORs for the risk of progression when using the LUS + DLco and LUS + PFT models*

	OR	95% CI	*P*
LUS + DLco model			
Difference in B‐lines	1.09	1.02–1.18	0.0185
Baseline B‐lines	1.11	1.04–1.22	0.0119
Difference in DLco%pred	1.03	0.941–1.13	0.529
LUS + PFT model			
Difference in B‐lines	1.08	1.01–1.18	0.0433
Baseline B‐lines	1.11	1.04–1.22	0.0136
Difference in DLco%pred	1.02	0.928–1.12	0.687
Difference in FVC%pred	1.04	0.926–1.18	0.524

* Overview of ORs for the parameters included in the LUS + DLco and LUS + PFT models. ORs represent the change in odds of radiographic progression per unit increase in the predictor. LUS variables are expressed per one B‐line increase. Pulmonary function variables are expressed per 1% predicted change. CI, confidence interval; DLco%pred, predicted diffusing capacity for carbon monoxide; FVC%pred, predicted forced vital capacity; LUS, lung ultrasonography; OR, odds ratio; PFT, pulmonary function test.

When using comparative C‐statistics, the LUS model did not have a significantly higher AUC when compared to the LUS + DLco model (*P* = 0.68), the LUS + PFT model (*P* = 0.588), the clinical model (*P* = 0.47), the PFT model (*P* = 0.079), and the symptom model (*P* = 0.067). The LUS + DLco model performed significantly better than the PFT model (*P* = 0.022) and the symptoms model (*P* = 0.020). There were no significant differences with the PFT + LUS model (*P* = 0.57), the clinical model (*P* = 0.23), and the LUS model (*P* = 0.68). The LUS + PFT model also outperformed the PFT model (*P* = 0.014) and the symptoms model (*P* = 0.013). No significant difference was found with the clinical model (*P* = 0.170), the LUS model (*P* = 0.587), and the LUS + DLco model (*P* = 0.571).

## DISCUSSION

Our findings suggest that changes in the number of B‐lines on LUS over time correlate with radiologic progression of ILAs in patients with RA. Although the correlation using the Likert scale showed a trend that did not reach statistical significance, a categorical approach to ILA progression, expressed as worsened, stable, and improved, showed a significant correlation. Patients who worsened radiologically on HRCT had a greater increase in B‐lines compared to those who remained stable, and a linear trend analysis supported a stepwise relationship. Although subgroup sizes were small and the CIs were very wide, the calculated odds ratios suggest that even modest increases in B‐lines may be associated with markedly higher odds of radiologic progression. This signal, however, did not extend when applied to large increases in B‐lines, probably due to a small number (n = 4) of patients in this subgroup. Furthermore, these data have to be interpreted with caution because the decreases in B‐lines also resulted in slightly elevated odds ratios, probably due to small sample sizes and possibly higher baseline B‐line counts.

In multivariable analyses, both the baseline number of B‐lines and the change in the number of B‐lines significantly predicted the chance of radiologic progression of ILAs. Models combining LUS with DLco or with full PFT achieved the highest discriminative power, although the added value over ultrasonography alone was not statistically significant. The model combining LUS and DLco significantly outperformed the models based on symptom scores and PFTs. There was a trend toward superior performance over the model based on high‐risk clinical characteristics (male sex, age, DAS28‐CRP, and presence of anti‐CCP antibodies), but this trend was not statistically significant. These results underscore the potential of LUS not only as a screening tool but also as a monitoring and prediction tool in RA‐ILA, while considering both baseline B‐lines and the change. We hypothesize that the presence of B‐lines at baseline likely reflects possible pre‐existing ILAs, indicating a higher inherent risk of progression.

Current European Respiratory Society (ERS) and American Thoracic Society (ATS) guidelines do not recommend LUS for the diagnosis or monitoring of ILD. Importantly, these guidelines primarily focus on established or clinically manifest ILD and do not specifically address the potential role of LUS in screening or longitudinal monitoring of subclinical disease in high‐risk populations such as patients with RA. In this context, our findings do not challenge existing recommendations but rather suggest a complementary role for LUS as an adjunctive screening or monitoring tool in selected patients with RA.

The main strength of this study is that it is the first study that shows that LUS might have a role in the assessment of an increase of radiologic changes or the appearance of new changes in a population of patients with RA. A major advantage of LUS is its dynamic bedside applicability. Furthermore, ultrasonography is already widely used in outpatient rheumatologic practice for the assessment of joints. Additionally, the absence of radiation makes this an excellent tool for serial follow‐up. Although ultrasonography remains an operator‐dependent technique, the interobserver and intraobserver agreement among the trained observers was robust and in line with previously published findings.^30^, ^31^ Nevertheless, further studies on repeatability and the learning curve of LUS are still needed to fully validate this tool and support its translation into clinical practice. Another major strength of this article is the longitudinal design including imaging, clinical, and lung function parameters and the use of a real‐world cohort of patients with RA.

Despite these strengths, several limitations must be considered. First, the results should be interpreted with caution given the limited sample size and very small subgroups. This contributed to wide CIs and limited statistical power, with some analyses revealing trends without reaching statistical significance. Nevertheless, these initial findings provide a solid foundation for future studies.

Second, after an initial dropout rate of 5% between intake and the first CT scan, there was an additional dropout of 14.3% for the one‐year time point. Dropout is an inherent challenge in prospective studies,^32^ and when asked about their reasons, patients cited the high burden of technical examinations, although this was not systematically assessed. This highlights the importance of ensuring these examinations are seamlessly integrated into routine clinical care and designed to take as little time as possible to maximize their effectiveness.

Third, the assessment of CT‐defined progression is subject to interobserver variability. In our study, interobserver agreement for both the presence and progression of ILAs was moderate to substantial, in line with previously published findings.^33^ To mitigate this limitation, all discrepancies were resolved using a consensus‐based approach. Furthermore, the use of a Likert scale represents a semiquantitative and observer‐dependent method for assessing radiologic progression. Although alternative scoring systems such as the Scleroderma Lung Study I (SLS‐I) score have been proposed, these were specifically developed and validated in systemic sclerosis–associated ILD (more homogeneous non‐specific interstitial pneumonia (NSIP) distributions). In contrast, RA‐ILD is characterized by greater heterogeneity in radiologic patterns, including a higher prevalence of usual interstitial pneumonia–like disease and a more patchy and asymmetric distribution, which may limit the applicability and validity of systemic sclerosis–derived scoring systems in this population. Quantitative CT approaches using artificial intelligence–based algorithms may offer more granular and objective assessments of fibrotic burden and represent an important future direction. However, these techniques require disease‐specific validation and standardized imaging protocols, which were beyond the scope of the present study but will be implemented in our follow‐up long‐term extension study.

Fourth, this is a single‐center, exploratory study. No correction for multiple testing was applied, and overinterpretation of results should be avoided.

Fifth, by including both baseline B‐lines and change in B‐lines in the same model, we adjusted for baseline, mitigating potential bias from regression to the mean. However, using raw change may still conflate true change with measurement noise. Additionally, the baseline scan was assessed by one ultrasonographer, whereas for the follow‐up LUS, approximately one‐fourth of scans were performed by a second reader, possibly also influencing the results even when the interobserver variability was substantial. Moreover, test‐retest reliability and sensitivity to change were not formally assessed, which limits conclusions regarding the reproducibility and responsiveness of the LUS measurements. Future studies could explore repeated‐measures or errors‐in‐variables models. Finally, external validation in larger cohorts is required to confirm our findings.

It is important to note that this article focused on the presence of radiologic progression and not clinical progression as defined by the ATS and INBUILD criteria. These results must be interpreted within this clinical context. Progression is currently being assessed on multiple facets, incorporating radiologic findings, PFT evolution, and changes in symptoms. LUS will most likely have to be placed in this multimodal context to evaluate progression by providing some valuable clues about possible subclinical involvement. It could play a role in justifying closer follow‐up or warranting further testing, potentially after risk stratification based on other risk factors.

To conclude, our findings suggest that LUS may aid in detecting radiologic progression and the appearance of new development of ILAs. This pilot study provides a foundation for future research to validate the observed correlation with radiologic progression. Further studies should explore how LUS can be integrated, most likely in a multimodal approach, to guide clinicians in monitoring ILA progression.

## AUTHOR CONTRIBUTIONS

All authors contributed to at least one of the following manuscript preparation roles: conceptualization AND/OR methodology, software, investigation, formal analysis, data curation, visualization, and validation AND drafting or reviewing/editing the final draft. As corresponding author, Dr Vermant confirms that all authors have provided the final approval of the version to be published and takes responsibility for the affirmations regarding article submission (eg, not under consideration by another journal), the integrity of the data presented, and the statements regarding compliance with institutional review board/Declaration of Helsinki requirements.

## Supporting information


**Disclosure Form**:

**Supplementary Figure 1.** Study flow diagram and analytic cohorts.
