# Supplementary material for: Lung Ultrasound for the Detection of Radiologic Progression of Interstitial Lung Abnormalities in Patients With Rheumatoid Arthritis
Source: Arthritis Rheumatol. 2026 May 3;78(11):2221–31. doi: 10.1002/art.70161 (PMC13712542; doi:10.1002/art.70161)
Supplement: Supplementary file 2 [file ART-78-2221-s001.docx]

### Suppelmentary Figure 1. Study flow diagram and analytic cohorts

Flow diagram illustrating patient inclusion and availability of imaging data. Of the 100 included patients, 2 were excluded prior to imaging and 3 did not undergo baseline HRCT, resulting in 95 patients with baseline HRCT. Follow-up HRCT was available in 81 patients, of whom 77 underwent per-protocol lung ultrasound (LUS) at follow-up.
